# Evaluation of literature searching tools for curation of mismatch repair gene variants in hereditary colon cancer

**DOI:** 10.1002/ggn2.10039

**Published:** 2021-02-18

**Authors:** Varun Kaushik, John‐Paul Plazzer, Finlay Macrae

**Affiliations:** ^1^ Melbourne Medical School The University of Melbourne Parkville Victoria Australia; ^2^ Department of Colorectal Medicine and Genetics, The Royal Melbourne Hospital Parkville Victoria Australia; ^3^ Department of Medicine, The Royal Melbourne Hospital The University of Melbourne Parkville Victoria Australia

**Keywords:** cancer, DNA mismatch repair, Lynch syndrome, genetic variation classification, database management systems, information storage and retrieval, literature searching

## Abstract

Pathogenic constitutional genomic variants in the mismatch repair (MMR) genes are the drivers of Lynch syndrome; optimal variant interpretation is required for the management of suspected and confirmed cases. The International Society for Hereditary Gastrointestinal Tumours (InSiGHT) provides expert classifications for MMR variants for the US National Human Genome Research Institute's (NHGRI) ClinGen initiative and interprets variants with discordant classifications and those of uncertain significance (VUSs). Given the onerous nature of extracting information related to variants, literature searching tools which harness artificial intelligence may aid in retrieving information to allow optimum variant classification. In this study, we described the nature of discordance in a sample of 80 variants from a list of variants requiring updating by InSiGHT for ClinGen by comparing their existing InSiGHT classifications with the various submissions for each variant on the US National Centre for Biotechnology Information's (NCBI) ClinVar database. To identify the potential value of a literature searching tool in extracting information related to classification, all variants were searched for using a traditional method (Google Scholar) and literature searching tool (Mastermind) independently. Descriptive statistics were used to compare: the number of articles before and after screening for relevance and the number of relevant articles unique to either method. Relevance was defined as containing the variant in question as well as data informing variant interpretation. A total of 916 articles were returned by both methods and Mastermind averaged four relevant articles per search compared to Google Scholar's three. Of relevant Mastermind articles, 193/308 (62.7%) were unique to it, compared to 87/202, (43.0%) for Google Scholar. For 24 variants, either or both methods found no information. All 6/80 (20%) variants with pathogenic or likely pathogenic InSiGHT classifications have newer VUS assertions on ClinVar. Our study demonstrated that for a sample of variants with varying discordant interpretations, Mastermind was able to return on average, a more relevant and unique literature search. Google Scholar was able to retrieve information that Mastermind did not, which supports a conclusion that Mastermind could play a complementary role in literature searching for classification. This work will aid InSiGHT in its role of classifying MMR variants.

**Abbreviations**
InSiGHT: International Society for Gastrointestinal Hereditary Tumours
LS: Lynch syndrome
MMR: mismatch repair
NCBI: US National Center for Biotechnology Information
NHGRI: US National Human Genome Research Institute
VUS: variant of unknown significance

## 1 INTRODUCTION

Lynch syndrome (LS) is the most common aetiology of hereditary colorectal neoplasia with a prevalence of 3% to 5% amongst colorectal cancer patients.^1^ LS is characterized by pathogenic variations in the DNA mismatch repair (MMR) genes (*MLH1*, *MSH2*, *MSH6* and *PMS2*), which are highly penetrant and confer an increased risk of colorectal cancer, amongst other cancers.^2^ Today, LS is often diagnosed with DNA sequencing technology which can identify pathogenic MMR variants. With advances in this technology and its widespread use in the diagnosis and management of LS and suspected cases, our awareness of novel variants in the MMR genes has increased dramatically. To deal with this influx, expert groups who specialize in particular syndromes for example, LS, have been formed to optimize classification for novel variants. The International Society for Hereditary Gastrointestinal Tumours (InSiGHT) provides expert classifications on MMR variants. InSiGHT's role has now also expanded to being recognized as a NHGRI ClinGen Variant Curation Expert Panel (VCEP) addressing the task of curation of MMR variants in the NCBI's ClinVar database. Relatedly, NCBI's ClinVar was launched in 2013 in an effort to increase variant data sharing and promote standardized classification of variants.

A key role of the InSiGHT VCEP is to reclassify variants on the ClinVar database whose genotype‐phenotype relationship is unclear or not definitive as understood by submitters. These include variants of uncertain significance (VUS); in addition, many variants receive discordant pathogenicity assignments when submitted to databases such as ClinVar. Discordance is multifactorial and variant interpretation is often generated from multiple sources, leading to a “silo effect,” whereby information is considered in isolation by different submitters. The result is a lack of centralized, contemporary information pertaining to a particular variant.^3^ Additionally, for the most complete picture about a particular variant to be gleaned, the submission of unpublished material and information may be encouraged. InSiGHT encourages the submission of unpublished clinical and research data by recognizing contributions through microattribution.^4^ InSiGHT at its Variant Interpretation Committee/VCEP Teleconferences frequently identifies critical unpublished information to aid interpretation. This information is documented on its database.

VUSs pose a particular clinical problem as they are not identified as benign with reference to the reference human genome reads, but a detrimental influence of the function of the gene is not apparent for them to be declared pathogenic on the basis of contemporary data. This leaves families carrying these variants in diagnostic limbo.^5^ For discordant variants, misclassification can result in serious clinical mismanagement across and within families, especially in the case where a variant is misclassified as benign and was later reclassified as pathogenic.^6^

To classify a variant, a biocurator may face a seemingly never‐ending literature search which may return many irrelevant results. Collecting information that is relevant to variant interpretation is an overwhelming manual task which will only become more onerous with the rate at which literature is now produced. However, there are now variant‐oriented search systems that could improve the quality of search results and by extension improve the efficiency of the curation process.^7^ These literature searching tools are able to find articles that mention specific variants using artificial intelligence and natural language processing. They have been purported to increase the yield of a literature search compared to traditional search methods.^7^ Whilst the literature does describe open source tools such as tmVar2.0 and LitVar which have been demonstrated to yield more articles than a standard PubMed search, the question as to whether these tools can be applied to a practical setting such as variant curation and interpretation remains unanswered.^8^, ^9^ For such tools to be useful, they would need to return articles that are relevant to the biocurator's task of classification. Such information includes experimental validation of variant functions, tumor and co‐segregation information, family history, in silico analysis and statistical methods to determine a probability of pathogenicity.

The literature so far has focused largely only on the correct identification of gene, mutation and disease within a paper by a literature searching tool.^9^ Furthermore, whilst there has been discussion of open source tools applied to breast and prostate cancer variants, analysis of specific applications of literature searching tools with MMR variants is mostly limited to a study which developed “Variation Annotation Schema” that aimed to capture important concepts and relations for human genetic variation.^10^ This schema was developed in response to the needs of InSiGHT biocurators and relates to the historical curation of the InSiGHT database and annotation of MMR genes. It was hoped it would provide a framework for future literature searching tools for MMR variants.

There now exist a range of commercially available literature searching tools and given the onerous task of manual curation, a tool that increases the efficiency or accuracy of the initial literature search could allow the optimum classification of MMR variants and could be beneficial in resolving discordant interpretation. We therefore set out to ask the question as to whether literature searching tools could add incremental value to the initial literature search to retrieve information for the classification of MMR variants submitted with different pathogenicity assignments.

## 2 AIMS AND HYPOTHESIS

Our first aim was to examine the nature of discordance in a set of MMR variants with different pathogenicity assignments, by comparing InSiGHT classifications to their associated assertions of pathogenicity on the ClinVar database. Our second aim was to identify the incremental value that could be added to an initial variant literature Google Scholar search that informs the MMR variant classification process by using the literature searching tool Mastermind. We hypothesized that amongst a sample of variants submitted to ClinVar with different pathogenicity assignments, Mastermind would add incremental value to the initial literature search for a variant being classified in the MMR classification process by providing a more relevant initial literature search and retrieve more unique information, compared to a standard Google Scholar search, for a particular variant. Given the importance of Lynch syndrome as the most common aetiology of hereditary gastrointestinal cancer, if literature searching tools could ease the burden on biocurators, then perhaps the promise of precision medicine could be more easily delivered by the more accurate classification of variants, and the resolution of discordant variants, which would ameliorate some of the associated clinical challenges and risks.

## 3 METHODS

### 3.1 Sample

In January 2020, a list of MMR variants with discordant classifications that require reviewing and updating on the ClinGen ClinVar database was provided to the InSiGHT VCEP by ClinGen as a part of InSiGHT's role in reclassifying these variants. This list was “prioritized” by ClinGen into several categories. The first of these were the “Alert” categories: which spanned variants that have existing InSiGHT classifications but now have more recent, differing classifications submitted to ClinVar from other non‐expert entities such as laboratories, familial cancer clinics or research institutions. Variants listed as “Priority” are those variants which do not necessarily have an InSiGHT classification, but now have newer, conflicting classifications on ClinVar from multiple submitters.

To describe the nature of discordance amongst this sample of variants (the first aim) and eventually use the sample to identify the value of Mastermind in an initial variant literature search (the second aim), it was important the sample reflected a typical situation that a biocurator may be faced with, that is: fulfilling the important role of the InSiGHT VCEP by reclassifying VUSs and resolving discordant variants. In order to address the two aims of this study, we used judgement sampling to identify which variants should be selected for inclusion in the study and this was on the basis of priority as designated by ClinVar and number of conflicting submissions on ClinVar. Judgement sampling refers to a sample chosen based on the prior knowledge of a subject and is useful for samples where the aim is to improve process performances, which in our case is the process of literature searching for MMR variant classification.^11^

We first focused our efforts on the “Alert” variants and then prioritized a selection of variants from the “Priority” group. We aimed for an arbitrary total of 80 variants which was thought to be a sufficient sample size to pilot the feasibility of Mastermind. As this was intended to be a study that examined the feasibility of using Mastermind across a range of different discordant settings, it was not deemed necessary (and was beyond the scope of this study) to test all of the variants in every category beyond “Alert”. As detailed statistics were not planned, there was no formal power calculation for sample size.

From the 80 variants on the list, all 31 variants in the 'Alert' category were selected for analysis on the basis of being of high priority (as designated by ClinGen) for InSiGHT to provide updated classifications. Further subgroups within the Alert category will be expanded upon in the Results section.

The remaining 49 variants were selected on the basis of multiple submissions with discordant interpretations by different submitters and were from the “Priority” category as designated by ClinGen. From the 'Priority' category, we focused on two sub‐groups. The first was variants that did not necessarily have an InSiGHT classification but had at least one conflicting pathogenic/likely pathogenic vs VUS/likely benign /benign submission from different sources on ClinVar, this being a medically significant conflict. Here we denote variants pooled across one or more InSiGHT classifications by a diagonal slash to denote OR (eg,: likely benign/benign) and conflicts of classification denoted by “vs” (eg, pathogenic/likely pathogenic vs VUS/likely benign/benign) To further prioritize these variants, we then derived the median number of submissions to ClinVar per variant, which was four, and selected all the variants with four or more submissions for inclusion. The first of these groups prioritized by this method contained 39 variants.

In the second subgroup of the “Priority” category, variants did not necessarily have an InSiGHT classification, but had at least one VUS and at least one likely benign/benign non‐expert panel classification on ClinVar. Since this group was deemed to be of lower priority and had a large number (540) of variants, we examined the top ten variants with the most assertions of pathogenicity on ClinVar.

### 3.2 The literature searching tool

The Mastermind Genomic Search Engine (Mastermind)^12^ was selected as the commercially available literature searching tool for comparison primarily because of its ease of use (as it does not require the use of Boolean search terms) and popularity amongst biocurators. Mastermind uses artificial intelligence, machine learning and genomic language processing to search the literature for gene variants. Such technology is able to identify the ways in which genes and variants are described in the literature and filter out erroneous information by incorporating knowledge of biology and human genomics. Mastermind is updated on a weekly basis. To maximize applicability of any results to a general setting we used the Basic, free edition of the software that simply required registration using an email address and password. To use Mastermind, a variant is entered into the search field (much like any Internet search engine) and Mastermind then returns all the articles it can find that mention the particular variant.

### 3.3 Standardizing Google Scholar search

The traditional searching method that we compared the results of Mastermind to was Google Scholar. Google Scholar's ability to search the full text of articles was the primary reason this was used as the standardized control over PubMed, which does not search full text. To standardize the Google Scholar search for comparison variants were entered into the Genomizer website interface^13^ which processes proteins and variants into the required search terms. For example for the variant c.1984A > C in *MLH1* (standardized nomenclature: NM_000249.3(MLH1):c.1984A > C (p.Thr662Pro)) Genomizer uses this to then generate a standardized Google Scholar search of: (MLH1 OR NM_000249.3 OR NM_000249) AND (c.1984A > C OR Thr662Pro OR T662P). Such a strategy captures the various ways in which the variant may be described in literature, allowing a standard Google Scholar search for a particular variant to be generated. The genes and variants were listed along with collected data and observations on an Excel spreadsheet.

### 3.4 Procedure/experimental protocol

In this study the independent variables were the search methodology (Google Scholar or Mastermind), the dependent variables were the data collected which were: number of articles retrieved for each search method per variant, number of relevant articles, number of articles unique to Mastermind or Google Scholar. The control to which Mastermind results were compared was Google Scholar. Other information collected on each of the variants related to gene information such as the gene name, variant, protein change, InSiGHT classification date, ClinVar submissions of pathogenicity and latest ClinVar submission date.

To address the second aim, whereby the potential value of Mastermind to the initial literature search for a variant was identified, all articles were screened for relevance. Relevance was defined by the author as containing the variant in question as well as any data informing the pathogenicity classification such as: tumor information, family history, co‐segregation data, in silico analysis, functional assays or statistical methods of predicting pathogenicity.

All 80 variants underwent both Google Scholar and Mastermind searches independently of each other. The procedure was as follows: The variant in question was taken from the ClinVar InSiGHT VCEP update list and entered into the Genomizer converter. A Google Scholar search term was generated from Genomizer; patents and citations were excluded from the search results as they were not searched by Mastermind. The number of articles returned was recorded and then subsequently each article was reviewed in full text and the variant mentions in each article scrutinized for relevance according to the definition above. Articles not in English were not counted as relevant articles and duplicates were only accounted for once.

A similar methodology was used for Mastermind whereby each variant was entered into the search interface and the total number of articles returned before and after screening for relevance was recorded. No advanced filters were applied as this required a commercial subscription, which may not be available to all biocurators. The articles returned by both methods were then viewed side by side and the number of articles unique to each search method was recorded. Statistical methods planned for this study were descriptive in nature and consisted of frequencies, means, medians and ranges. Further statistical analysis including testing formally the hypothesis that: Mastermind's results would be more relevant or contain more unique information than Google Scholar's, was deemed beyond the scope of a limited feasibility study that was not randomized nor blinded.

### 3.5 Ethics

Our study met the criteria for a quality assurance study in the Department of Colorectal Medicine and Genetics, The Royal Melbourne Hospital (RMH), Melbourne, Australia. The Office for Research, RMH granted the reference number QA2020043.

### 3.6 Data availability

All current variant classifications are openly available at ClinVar and InSiGHT. Data generated by this project are included as Supplementary Table 1.

## 4 RESULTS

### 4.1 The nature of discordance amongst variants selected for inclusion

#### 4.1.1 Genes and corresponding variants selected for inclusion in the study

The 80 variants across the genes *MLH1, MSH2, MSH6 and PMS2* were examined as described in Table 1. *MLH1* and *MSH2* variants were most common each representing approximately one third of the sample, with *PMS2* variants being the least common.

**TABLE 1:** Frequency of variants examined by gene (n = 80 variants)

Gene	Frequency (%)
*MLH1*	24 (30.0)
*MSH2*	25 (31.3)
*MSH6*	20 (25.0)
*PMS2*	11 (13.8)

#### 4.1.2 Discordance of InSiGHT assertions of pathogenicity compared to ClinVar assertions of pathogenicity

Of the 80 variants, 16/80 (20%) were classified by InSiGHT as being pathogenic or likely pathogenic. All 16 InSiGHT pathogenic/likely pathogenic variants had only conflicting VUS assertions on the ClinVar database, for which there were 32 assertions.

For the 38/80 (47.5%) of variants that were classified as VUS, by InSiGHT, 23 variants (28.8%) had conflicting likely pathogenic/pathogenic assertions. Amongst these 23, 10 variants had only likely pathogenic/pathogenic assertions on the ClinVar database, with the remaining 13 recording a mixture of likely pathogenic/pathogenic assertions and VUS assertions. 15/80 (18.8%) of InSiGHT VUS variants had conflicting likely benign/benign assertions. Amongst these 15, five variants had only likely benign/benign conflicting.

assertions. The remaining 10 InSiGHT VUS variants had a mixture of likely benign/benign assertions and VUS assertions. For the 26/80 variants that were not classified by InSiGHT, all 26 variants had a mixture of conflicting likely pathogenic/pathogenic and VUS assertions.

Table 2 shows the frequency of the different ClinVar assertions of pathogenicity for each of the variants selected, organized by their InSiGHT classification. Whilst InSiGHT provides one classification per variant, ClinVar accepts multiple assertions of pathogenicity from multiple submitters per variant with 357 ClinVar assertions across the 80 variants selected in the study. For the 16 InSiGHT pathogenic/likely pathogenic variants, there were 32 assertions on the ClinVar database. For the 38/80 (47.5%) of variants that were classified as VUS by InSiGHT, 108/208 (51.9%) had ClinVar VUS assertions. However, additionally, 55/208 (26.4%) were ClinVar likely benign/benign and 45/208 (21.6%) were ClinVar pathogenic/likely pathogenic. The remaining 26 variants in the study that were not classified by InSiGHT had 117 assertions of pathogenicity on the ClinVar database. 47/117 (40.2%) assertions were pathogenic/likely pathogenic and the majority were VUS assertions with 70/117 (59.8%) assertions.

**TABLE 2:** ClinVar assertions of pathogenicity for the 80 variants organized by InSiGHT classification

	ClinVar Assertions of Pathogenicity		
	ClinVar Pathogenic/Likely Pathogenic	ClinVar VUS	ClinVar Likely Benign/Benign
InSiGHT Pathogenic/Likely Pathogenic (n = 16/80 variants)	0	32	0
InSiGHT VUS (n = 38/80 variants)	45	108	55
InSiGHT Not classified (n = 26/80 variants)	47	70	0

*Note*: NB: *“*n = 16/80” indicates that 16 OF a total of 80 variants received a given classification or classifications. “Pathogenic/Likely Pathogenic” indicates the variants were classified as either Pathogenic OR Likely Pathogenic.

### 4.2 The incremental value of mastermind in the variant literature search

#### 4.2.1 Total yield and relevance of Google Scholar and Mastermind searches

Table 3 demonstrates that searches in Google Scholar and Mastermind across the 80 variants yielded 477 and 439 articles respectively, giving a total of 916 articles screened for relevance. Per search, Google Scholar on average yielded six articles, compared to Mastermind which on average yielded five articles. However, when screened for relevance, a greater proportion of Mastermind articles (308/429, 70.2%) were deemed relevant when compared to the control, Google Scholar (202/477, 42.3%). Per search, Mastermind yielded more relevant articles on average from the original search when compared to Google Scholar control searches with means of approximately four articles and three articles respectively.

**TABLE 3:** Number of articles yielded by Google Scholar and Mastermind

	Number of articles ‐ GS (control)^a^	Number of articles ‐ MM^2^	Number of relevant articles – GS (control) (% of total)	Number of relevant articles – MM (% of total)
Total	477	439	202 (42.3)	308 (70.2)
Mean (per search)	5.96	5.49	2.53	3.89
Median (per search)	3.5	2.5	1.0	2.0
Range (per search)	0‐50	0‐63	0‐19	0‐32
Discordant assertions				
InSiGHT Pathogenic/Likely Pathogenic vs Newer ClinVar VUS/Likely Benign (n = 16 variants)	57	34	34 (59.6)	32 (91.3)
InSiGHT VUS vs Newer ClinVar Pathogenic/Likely Pathogenic (n = 10 variants)	32	49	12 (37.5)	49 (89.8)
InSiGHT VUS vs Newer ClinVar Likely Benign/Benign (n = 5 variants)	21	78	14 (66.7)	15 (19.2)
ClinVar Pathogenic/Likely vs ClinVar Likely Benign^a^ (n = 39 variants)	249	173	89 (34.9)	119 (68.8)
ClinVar VUS vs ClinVar Likely Benign/Benign^b^ (n = 10 variants)	118	93	55 (46.6)	88 (94.6)

Abbreviations: GS, Google Scholar, MM, Mastermind.

a Some variants in this category were yet to be classified by InSiGHT but were in the scope of the InSiGHT Variant Curation Expert Panel (VCEP). They had at least one Pathogenic/Likely Pathogenic ClinVar assertion and at least one VUS/Likely Benign/Benign ClinVar assertion (medically significant conflict).

b Variants in this category were not classified by InSiGHT but were in the scope of the InSiGHT VCEP. They had at least one VUS ClinVar assertion and at least one Likely Benign/Benign assertion.

#### 4.2.2 Unique articles for Google Scholar and Mastermind searches

The number relevant articles that were unique to either Google Scholar or Mastermind can be found in Table 4. Mastermind found an increased proportion of relevant articles that were unique when compared to Google Scholar (193/308, 62.0% vs 87/202, 43.0%). Additionally, per search, Mastermind had an average of two unique articles, compared to one for Google Scholar. By ClinVar category of discordance, Mastermind returned more unique search results in every category.

**TABLE 4:** Unique number of articles across Google Scholar and Mastermind Searches

	Number of relevant articles unique to GS (control) (% of GS relevant articles)	Number of relevant articles unique to MM (% of MM relevant articles)
Total	87 (43.0)	193 (62.7)
Mean (per search)	1.09	2.41
Median (per search)	0.00	1.00
Range (per search)	0–7	0‐20
Discordant assertions		
InSiGHT Pathogenic/Likely Pathogenic vs Newer ClinVar VUS/Likely Benign (n = 16 variants)	19 (55.9)	27 (64.2)
InSiGHT VUS vs Newer ClinVar Pathogenic/Likely Pathogenic*/*n = 10 variants)	4 (33.3)	36 (81.8)
InSiGHT VUS vs Newer ClinVar Likely Benign/Benign (n = 5 variants)	5 (35.7)	6 (40.0)
ClinVar Pathogenic/Likely vs ClinVar New Likely Benign^a^ (n = 39 variants)	36 (41.4)	68 (57.1)
ClinVar VUS vs Likely Benign/Benign^b^ (n = 10 variants)	23 (41.8)	56 (63.6)

a Some variants in this category were yet to be classified by InSiGHT but were in the scope of the InSiGHT Variant Curation Expert Panel (VCEP). They had at least one Pathogenic/Likely Pathogenic ClinVar assertion and at least one VUS/Likely Benign/Benign ClinVar assertion (medically significant conflict).

b Variants in this category were not classified by InSiGHT but were in the scope of the InSiGHT VCEP. They had at least one VUS ClinVar assertion and at least one Likely Benign/Benign assertion.

#### 4.2.3 Instances where Google Scholar or Mastermind returned no information

Table 5 demonstrates that there was a total of 24 instances where either one or both search method returned 0 results. A key finding here is that there were 14 variants for which only one of the two searching methods (seven each) identified articles ‐ almost one in five variants (17.5%) have missed articles in one of the search methods. Additionally, there were 10 variants for which neither search method found any information.

**TABLE 5:** Number of variants for which search methods found no information (n = 24)

Category	Frequency (%)
Google Scholar found articles, Mastermind did not	7 (29%)
Google Scholar did not find articles, Mastermind did	7 (29%)
Neither Google Scholar nor Mastermind found any articles	10 (42%)
Total	24

## 5 DISCUSSION

### 5.1 The nature of discordance in the sample of MMR variants

The first aim of our study was to describe the nature of discordance amongst variants that were known to be discordant, which would ultimately be used to compare Mastermind to Google Scholar. Of the 80 variants, 16/80 (20%) of variants that were classified by InSiGHT as pathogenic or likely pathogenic now have newer, recent assertions as a VUS on ClinVar, with such assertions tending to be from the following 12 months since the variant was last evaluated by an expert panel. As such, even with recent expert evaluation, conflicting information must be dealt with on a rolling basis. Literature searching tools may be able to aid with this, as will be discussed later in this article.

Additionally, while none of the variants in the study had benign/likely benign classifications by InSiGHT, those that were classified as VUS by InSiGHT did indeed have a substantial number of benign/likely benign classifications on ClinVar, with 55/208 of the assertions being of this nature in ClinVar and another 45 as pathogenic/likely pathogenic. However, the most significant features of discordance were newer VUS assertions in the setting of a previously pathogenic InSiGHT classification and the emergence of benign/likely benign classifications by ClinVar submitters that were classified as VUS by InSiGHT. The sample of variants used for determining the utility of literature searching tools in the initial literature search likely reflected a typical setting in which literature searching tools are of most use, that is, amongst variants with discordant classifications and in particular where newer classifications are VUSs. This could inform future work of the InSiGHT VCEP as it works to resolve discordant classifications and reclassify variants of unknown significance, notably because such discordant interpretations may have serious clinical consequences.

### 5.2 The proposed value of Mastermind in a variant literature search

Our second aim was to identify the incremental value that a literature searching tool may add to the initial literature search for the classification of MMR variants. To identify this, we examined overall yield and relevance of Mastermind searches and compared them to Google Scholar results to see whether Mastermind would provide a more relevant initial literature search with a greater proportion of unique information. Whilst Google Scholar results initially returned more articles, after screening each article for relevance, Mastermind returned a greater proportion of articles that were relevant, across most categories of discordance. Whilst this study did not seek to reclassify variants on the basis of the results of the differing search methods, in terms of incremental value added to the classification process, an initial variant search through Mastermind may be more useful than a traditional Google Scholar search. One could infer that this would allow the biocurator to find more actionable information per search, thereby allowing optimal classification. The biocurator may stop searching after the more efficient Mastermind search, as sufficient evidence might have been gleaned to allow definitive classification.

Another important aspect to consider in the value of literature searching tools is whether they find information not found by traditional methods. To identify this, the relevant articles that were unique to Google Scholar or Mastermind were recorded. In total, 87/202 (40.3%) of relevant Google Scholar articles were unique to Google Scholar, with an average of one unique, relevant article per search. On the other hand, 193/308 (62.0%) of Mastermind relevant articles were unique to Mastermind. Mastermind searches averaged two unique, relevant articles per search. When one considers that the overall average yield for Mastermind before screening for relevance was four articles, this suggests that a substantial proportion of total information found by Mastermind was unique. These results suggest that in addition to returning more relevant results, Mastermind was able to add significantly to a Google Scholar search by finding information that would have otherwise not been found. However, 40.3% of relevant Google Scholar articles were also unique ‐ which points to the continued currency of Google Scholar and traditional searching methods. In terms of the incremental value in the classification process of discordant variants, missing information from unique articles may be more recent or may not have been found by previous evaluators and may contain information key to resolving conflicting classifications. These articles would not have been retrieved if the relevant searching method was not used. This work suggests that for literature‐based curation, Mastermind and Google Scholar can be used in conjunction to achieve a more comprehensive literature search.

Another measure useful in examining the potential value of Mastermind was cases where either search method returned no information at all. This is a significant metric if one considers that given the sheer volume of information available, a true zero result may point convincingly to the variant being novel if it is not also on public variant databases. It is possible that an article pertaining to a variant could be published in the literature and its containing article missed by all search methods ‐ which may be the case for academic submission ‐ however it was beyond the scope of this work to explore this possibility. We sought to see whether Mastermind could find additional information when Google Scholar could not find any information, which might warrant the further use of Mastermind in initial searching. There were 24 variants for which either both or one search method did not return any information. Of these 24, in the case of 10 of them, neither Google Scholar nor Mastermind retrieved any articles ‐ suggesting that very limited data exists for these 10. There were 14 for which only one of the searching strategies (seven each) identified information, which points to Mastermind having a complementary role in the initial literature searching strategy for variant classification.

### 5.3 Mastermind in the context of previous work on literature searching tools and future directions

To our knowledge, this is the first study that uses a sample of discordant MMR variants and attempts to identify the incremental value that commercial literature searching tools might add to the process of retrieving information related to classification of MMR variants.

Our findings are consistent with the existing opinion on literature searching tools in the MMR space: that literature searching tools, whilst not replacing traditional searching methods, can serve a complementary role in the biocurator's toolkit.^10^ Where our study differs from previous work is primarily on the basis of methodology. In our study, Google Scholar tended to return a greater overall search than Mastermind which is contrary to prevailing conclusions in other papers: that automated literature searching tools yield a greater number of publications when compared to traditional search methods.^9^ This discrepancy is likely because most papers benchmark to PubMed, which does not search full text, but rather title and abstract. One 2010 study estimated that only 30% of all protein‐protein interactions are mentioned in the title and abstract, which PubMed searches are limited by.^14^ As such we used Google Scholar which can search full text, which is what Mastermind (and many other emerging literature searching tools) can do. The literature also tends to focus on assessing literature searching tools on the basis of their ability to find natural language paired with mutation mentions that is, the simple occurrence of information within a particular article and seldom describe the relevance of the information that surrounds the mutation mention.^15^

Instead, we sought to assess Mastermind on the basis of screening each article for information relevant to a biocurator reclassifying discordant variants or VUSs. In this study, tables and figures were searched to locate information on family history, co‐segregation data, summaries of in silico prediction and other functional information. Such information can be interpreted beyond the main aim or purpose of the article in question. In general, most variant strings/mentions tended to be in the Tables, Figures and/or Results. Previous studies have limited their application of literature searching tools to ones that have been designed primarily for research purposes, whereas our study uses a commercial one applied to a specific purpose, that is, literature searching for discordant MMR variant interpretation.^8^ In the MMR space, previous work by Verspoor et. al developed a Variant Annotation Schema which was hoped to be the basis of future literature searching tools.^10^ This study supports this work by showing the potential value of a commercially available literature searching tool used in the literature search for variants in the setting where information directly related to classification is sought.

In addressing generalizability, our first aim established that our sample contained a significant number of variants with newer pathogenic/likely pathogenic or likely benign classifications on the ClinVar database than their existing InSiGHT classifications. Being varied in discordance presents a typical setting in which a biocurator may use literature searching tools to conduct an initial literature search to classify discordant variants.^16^ An increase in sample of variants would improve generalizability of these results and other searching tools could be trialed to explore the utility of such tools other than Mastermind. Limitations of this study lie in the fact that it was a single investigator study; in the future the likely inter‐operator variability of searching could be addressed by deploying more investigators. Further extensions with the use of F‐measures and statistical hypothesis testing methods may make this work more comparable to the existing literature.^17^ In terms of future directions, one may attempt to address processes beyond the initial literature search to assess whether information found by literature searching tools was later actively used in formal classification of variants by groups such as the InSiGHT VCEP. The current work will usefully inform the work of the InSiGHT VCEP as it works to reach a consensus on the pathogenicity of the discordant variants studied here. Structured interviews with biocurators may be helpful in quantifying their opinions on emerging literature searching tools, as the literature only points to a small survey of 30 biocurators in 2012, which, given the emergence newer commercially and non‐commercially available searching tools such as Varsome, may be outdated.^18^

## 6 CONCLUSION

Our study has showed that for a sample of MMR variants with discordant classifications, Mastermind added incremental value to the initial literature search for a variant in question by providing a greater proportion of relevant articles overall and on average per search. We identified that Mastermind presented a greater proportion of unique articles not found by a Google Scholar search, highlighting its potential to source information missed by traditional searching methods. Given Mastermind still missed some information, it would not completely replace Google Scholar, but would be a very useful, complementary feature in the biocurator's variant interpretation toolkit.

Optimal MMR variant classification relies on the biocurator not only being able to retrieve a comprehensive literature search but also accessing information identified as relevant to the purpose of variant classification. The literature search should also not miss key information that might hold important answers related to optimal classification. Being an onerous task, if literature searching tools are able to add value to the initial search process and hence the overall classification process, then one may ultimately be able to resolve discordant interpretations and reclassify VUSs more efficiently and more accurately. The InSiGHT VCEP is committed to this task and delivering on the promise of precision medicine for patients and their families where it is hoped that literature searching tools may play a valuable role in this effort.

## CONFLICT OF INTEREST

The authors have had no role in Mastermind, Genomenon or Google and have no conflicts of interest to disclose.

## AUTHOR CONTRIBUTIONS

**Varun Kaushik:** Data curation, Formal analysis, Investigation, Methodology, Project administration, Writing‐original draft, Writing‐review & editing. **John‐Paul Plazzer:** Conceptualization, Data curation, Methodology, Software, Supervision, Validation, Writing‐review & editing. **Finlay Macrae:** Conceptualization, Investigation, Methodology, Project administration, Resources, Supervision, Validation, Writing‐review & editing.

### PEER REVIEW

The peer review history for this article is available at https://publons.com/publon/10.1002/ggn2.10039.

## Supporting information


**Appendix** S1: Supporting Information

## Data Availability

The data that supports the findings of this study are available in the supplementary material of this article.
