# Supplementary material for: Evaluation of literature searching tools for curation of mismatch repair gene variants in hereditary colon cancer
Source: Adv Genet (Hoboken). 2021 Feb 18;2(1):e10039. doi: 10.1002/ggn2.10039 (PMC9744508; doi:10.1002/ggn2.10039)
Supplement: Supplementary file 1 — Appendix S1: Supporting Information [file GGN2-2-e10039-s001.zip › AdvGenet_0028_Kaushik_TPR_ACC.docx]

**Evaluation of literature searching tools for curation of MMR gene variants in hereditary colon cancer**

Varun Kaushik^*^, John Paul Plazzer, Finlay Macrae

*Corresponding

| Review timeline: | Date Submitted: 28-Sep-2020  Editorial Decision: 05-Oct-2020 Minor Revision prior to review  Revision Received: 02-Nov-2020  Editorial Decision: 14-Dec-2020 Accept in Principle  Revision Received: 12-Jan-2021  Accepted: 13-Jan-2021 |
| --- | --- |

Editor: Myles Axton

| Initial Editorial Evaluation 05-Oct-2020 |
| --- |

We think it would be great for Advanced Genetics in terms of scope. We think three minor changes would make it easier for peer reviewers to understand and increase its utility to the community. Please don't worry about formatting for initial peer review. Just give full methods and full references with all names and titles, and include the data from your searches in supplementary files. 

[1. What if there is only hay, no needles? If the problem is the uncertain information associated in specific variants that need expert review in ClinVar because of new reports of incomplete provenance and with conflicting evaluation by poorly documented clinical testing services, will searching for those same variants in the published literature increase the consensus and accuracy of expert review, or are other sources apart from publications needed?] 
[2. How does context help, and do we need a separate search? What is the value of following up Mastermind or Google Scholar search with the context in which the variant string is found? Are Tables (and Supplementary Tables) in publications more useful than the assertions found in the main Results, Abstract and Discussion of publications (uses for context AI such as Scite AI or Semantic Scholar?)] 
[3. Show the data! Include Supplementary Tables with the actual results used here, references, context, evaluation of the assertions for their semantic precision and transparency, and their suitability to the biocurator or InSiGHT expert in adding to or refining the variant’s risk score in ClinVar.

| 1^st^ Peer Review 02-Nov-2020 to 14-Dec-2020 |
| --- |

**Reviewer #1**

This is a very important and useful method. It will with no doubt be very useful for the VUS committee when reviewing MMR variants.

1.1 It would improve the manuscript if the authors could provide a more user-friendly description of the method. It is quite difficult to follow for non-AI people.

**Reviewer #2**

The manuscript by Varun et al. addresses the important issue of discrepancies in MMR gene variant classifications and the potential usefulness of different literature search tools to retrieve information relevant to the clinical interpretation process. The different combinations of conflicting interpretations provided by the InSiGHT expert panel and ClinVar submitters are described for a selected list of 80 variants prioritized for assessment based on clinical relevance. Then, the number and information contents (relevant or not to the interpretation process) are compared between a traditional literature search tool, Google Scholar, and a recently developed Genomic Search Engine, Mastermind Genomenon. While Mastermind has a better performance, the two approaches are shown to complement each other, indicating the importance of using both at this time.
The manuscript is well written, the aims are clearly stated and the methodology is sound. Since this is a proof of principle project, descriptive statistics is applied. 
Here are a few comments with suggestions that might be considered to improve the manuscript and clarify some issues:

2.1-The first aim of the study is to describe the nature of discordance in a sample of 80 selected variants. However, while judgement sampling was used to identify the most clinically relevant variants, a more thorough description could be given in order to view the full landscape of conflicting interpretations between the InSiGHT/ClinGen VCEP and ClinVar submissions or within the latter only. For instance, how many variants overall have a VUS vs likely benign/benign classifications (considering InSiGHT vs ClinVar as well as those not assessed by InSiGHT)? No variants with InSiGHT benign/likely benign classification are considered, but it would be interesting to see the numbers of discordant assertions could be specified also for these, and so on for other combinations. This is important also to have an estimate of the overall fractions of the different existing types of discrepancies, compared to those selected for the study.  

2.2-It would be useful to specify the time frame of the “newer” ClinVar assertions versus existing InSiGHT classifications (date of InSiGHT classifications vs time of the new ClinVar assertions). 

2.3-On page 11, second paragraph (and Table), it would be useful to know whether (and how many, if any) InSiGHT VUS had both likely benign/benign and pathogenic/likely pathogenic ClinVar assertions. Otherwise, it should be specified that there was no instance of this type of discrepancy. In the same paragraph, the 117 assertions mentioned in the sentence before the last one should be total (and not pathogenic), pathogenic (47) and VUS numbers are specified thereafter.

2.4-It seems that literature data did not lead to class changes for the 80 variants considered in the study. This might be a limit of the literature approach, and should be discussed. Is it possible that the retrieved papers had already been considered by InSiGHT panelists and/or ClinVar submitters? Could the authors provide data on overlap between retrieved literature and that used by InSiGHT or reported by ClinVar submitters? It would be interesting to show and discuss a few examples of variants (ie, one for each subgroup), describing the types of discrepancies and the specific information provided by the search engines for them compared to existing variant specific data.

**Reviewer #3**

The article is well written and best serves biocurators faced with the daunting task to collect specific variant information for the most informed pathogenicity assessment. The authors address the retrievability and segmentation of important information used for the expert review of the clinical significance of potentially disease-causing variations, the numbers of which have drastically increased. The nature of discordance amongst variant classifications is of importance to a broader audience as are the tools available. This is of particular utility for Lynch syndrome variant classification as thorough understanding of the spectrum and complexity of variant information already exists through ongoing variant pathogenicity classification efforts while the dangers of misclassification or ‘diagnostic limbos’ remain a concern.  

The authors compare the article yield, relevance and uniqueness obtained by two search approaches (Google Scholar and Mastermind) of 80 variants, showing that while both methods retrieve relevant unique articles, Mastermind did so more frequently. The authors show that literature searching tools can add incremental value to manual curation, to help decrease classification discordance or absence across a set of MMR variants. While facilitating this process is extremely valuable in helping reduce variant classification discordances, the link to delivering the promise of precision medicine is perhaps not within the deliverables of literature searching tools without further discussion.  

More specific comments: 

3.1 Although it is clear that both methods resulted in unique relevant articles and hence complement each other well, it is unclear why the authors conclude Mastermind to play a complementary role to the standard Google Scholar (control), not the other way around (although discussed). While both search methods missed some information, does the higher number of search results by Google Scholar outweigh the higher number of relevant articles with a higher number and percentage of unique articles yielded by Mastermind or has this been misunderstood? 

3.2 For clarity, it would be interesting to include the authors affiliation or lack of it to the chosen commercial literature searching tool. 

3.3 Duplicate search results were included only once, but when assessing articles for relevance, were duplicate findings also ruled out (ie. Original articles included only?)

3.4 If feasible, it would be interesting to know whether articles not found by either method for 10 variants exist in English.

3m1 Page 8. Second last line: “towas”

3m2 There are no ethical concerns or lack of analysis methods etc.

| 1^st^ Editorial Decision 14-Dec-2020 |
| --- |

**Editorial decision:** Accept in principle, provided the revision incorporates the minor revisions recommended by all reviewers

**Editor’s understanding of the reviews**

**Reviewer 1** recommends Minor revision

The reviewer is supportive but suggests that the methods be more clearly explained for a range of users.

**Reviewer 2** recommends Minor revision

The reviewer is engaged and suggests the analysis be made more useful in evaluation of VUS by incorporating analysis of the timescale of variant reevaluation in databases as well as the relative performance of these tools in identifying articles that help with VUS reevaluation in the case of discordant labels.

**Reviewer 3** recommends Accept

The reviewer is engaged and poses critical questions that should be addressed

| Author’s Response to 1^st^ Review 12-Jan-2021 |
| --- |

| **Suggested Title: Evaluation of literature searching tools for curation of MMR gene variants in hereditary colon cancer**  These are the main reviewer recommendations that the editors believe will make the biggest improvement to this article. **Please do address all reviewer comments listed in the decision letter in your point-by-point response** (you may continue this table to do so if you wish). We hope this summary helps you to understand our decision and expedites the revision process. We value feedback from author and referees alike.  [AdvGenet@wiley.com](mailto:AdvGenet@wiley.com) | | | |
| --- | --- | --- | --- |
| **Referee comments Reviewer comments** | **Editor recommendation** | **Author reply** | **Changes to Manuscript** |
| 3.2 For clarity, it would be interesting to include the authors affiliation or lack of it to the chosen commercial literature searching tool. | ED1 Please declare in the conflict of interest statement explicitly whether or not any of the authors has or has had any role in Mastermind or Google | None of the authors had any role in Mastermind or Google. | Conflict of interest / disclosures have been added to the end of the manuscript, detailing the previous. |
| 1.1 It would improve the manuscript if the authors could provide a more user-friendly description of the method. It is quite difficult to follow for non-AI people. | ED2 The readership will be clinicians and biocurators, so make clear what analytic decisions were made and why. | As it is hoped that this article serves as pilot project/introduction, the authors agree that the subject material may complex to a more general audience. | Some of the language in the Methods surrounding filters and artificial intelligence has been simplified or explained. |
| 2.1 how many variants overall have a VUS vs likely benign/benign classifications (considering InSiGHT vs ClinVar as well as those not assessed by InSiGHT)? No variants with InSiGHT benign/likely benign classification are considered, but it would be interesting to see the numbers of discordant assertions could be specified also for these, and so on for other combinations. This is important also to have an estimate of the overall fractions of the different existing types of discrepancies, compared to those selected for the study.  2.3 On page 11, second paragraph (and Table), it would be useful to know whether (and how many, if any) InSiGHT VUS had both likely benign/benign and pathogenic/likely pathogenic ClinVar assertions. Otherwise, it should be specified that there was no instance of this type of discrepancy. In the same paragraph, the 117 assertions mentioned in the sentence before the last one should be total (and not pathogenic), pathogenic (47) and VUS numbers are specified thereafter. | ED3 Give numbers for the overall landscape of variants, highlighting conflicting classifications. | We agree that it is useful to describe the overall landscape of variants and highlight the conflicting classifications at a variant level – in addition to providing analysis at an assertion level. | A paragraph detailing the landscape of variants has been inserted into the Results section 3.1.2. |
| 2.2-It would be useful to specify the time frame of the “newer” ClinVar assertions versus existing InSiGHT classifications (date of InSiGHT classifications vs time of the new ClinVar assertions).  2.4-It seems that literature data did not lead to class changes for the 80 variants considered in the study. This might be a limit of the literature approach, and should be discussed. Is it possible that the retrieved papers had already been considered by InSiGHT panelists and/or ClinVar submitters? | ED4 Include analysis of the dates of classification and discuss whether discrepancies are due to speed of updating. Given the slowness of publication relative to curation, what is your recommendation with respect to the limits of literature-based curation? | Discrepancies can be due to speed of updating (i.e if new conflicting information is published after an Expert Panel evaluation). Given the significant resources required for expert panel evaluation, using both Mastermind and Google Search may allow for a more comprehensive literature search to retrieve information to be brought for evaluation. | Clarification of this has been provided in the discussion section 4.1 |
| 3.1 Although it is clear that both methods resulted in unique relevant articles and hence complement each other well, it is unclear why the authors conclude Mastermind to play a complementary role to the standard Google Scholar (control), not the other way around (although discussed). While both search methods missed some information, does the higher number of search results by Google Scholar outweigh the higher number of relevant articles with a higher number and percentage of unique articles yielded by Mastermind or has this been misunderstood?  3.3 when assessing articles for relevance, were duplicate findings also ruled out | ED5 What is the value of unique articles? Should not the method that yields the highest number of articles be recommended as the best approach? Why is Google still the recommended method rather than using both and de-duplicating the articles? | Unique articles are articles that would have been otherwise missed had the relevant searching method not been used. | Clarification of this point has been made in the discussion section 4.2 |
| 3.4 If feasible, it would be interesting to know whether articles not found by either method for 10 variants exist in English. | ED6 What is the best way to evaluate known articles reporting variants that cannot be found by either method? | Articles may be absent from search engines because they are missed by indexing. Mastermind indexes weekly. Articles that do not appear on traditional search methods or that are picked up by Mastermind may commonly be academic work such as a PhD thesis. This work can contain valuable functional information. Verification of such information may be achieved by contacting the relevant submitter/author. However, this possibility was not explored in this work as it was considered beyond the scope of the paper. | Change to manuscript added in 4.2 discussion |

**Reviewer #1**

This is a very important and useful method. It will with no doubt be very useful for the VUS committee when reviewing MMR variants.

1.1 It would improve the manuscript if the authors could provide a more user-friendly description of the method. It is quite difficult to follow for non-AI people.

**Reviewer #2**

The manuscript by Varun et al. addresses the important issue of discrepancies in MMR gene variant classifications and the potential usefulness of different literature search tools to retrieve information relevant to the clinical interpretation process. The different combinations of conflicting interpretations provided by the InSiGHT expert panel and ClinVar submitters are described for a selected list of 80 variants prioritized for assessment based on clinical relevance. Then, the number and information contents (relevant or not to the interpretation process) are compared between a traditional literature search tool, Google Scholar, and a recently developed Genomic Search Engine, Mastermind Genomenon. While Mastermind has a better performance, the two approaches are shown to complement each other, indicating the importance of using both at this time.
The manuscript is well written, the aims are clearly stated and the methodology is sound. Since this is a proof of principle project, descriptive statistics is applied. 
Here are a few comments with suggestions that might be considered to improve the manuscript and clarify some issues:

2.1-The first aim of the study is to describe the nature of discordance in a sample of 80 selected variants. However, while judgement sampling was used to identify the most clinically relevant variants, a more thorough description could be given in order to view the full landscape of conflicting interpretations between the InSiGHT/ClinGen VCEP and ClinVar submissions or within the latter only. For instance, how many variants overall have a VUS vs likely benign/benign classifications (considering InSiGHT vs ClinVar as well as those not assessed by InSiGHT)? No variants with InSiGHT benign/likely benign classification are considered, but it would be interesting to see the numbers of discordant assertions could be specified also for these, and so on for other combinations. This is important also to have an estimate of the overall fractions of the different existing types of discrepancies, compared to those selected for the study.  

2.2-It would be useful to specify the time frame of the “newer” ClinVar assertions versus existing InSiGHT classifications (date of InSiGHT classifications vs time of the new ClinVar assertions). 

2.3-On page 11, second paragraph (and Table), it would be useful to know whether (and how many, if any) InSiGHT VUS had both likely benign/benign and pathogenic/likely pathogenic ClinVar assertions. Otherwise, it should be specified that there was no instance of this type of discrepancy. In the same paragraph, the 117 assertions mentioned in the sentence before the last one should be total (and not pathogenic), pathogenic (47) and VUS numbers are specified thereafter.

2.4-It seems that literature data did not lead to class changes for the 80 variants considered in the study. This might be a limit of the literature approach, and should be discussed. Is it possible that the retrieved papers had already been considered by InSiGHT panelists and/or ClinVar submitters? Could the authors provide data on overlap between retrieved literature and that used by InSiGHT or reported by ClinVar submitters? It would be interesting to show and discuss a few examples of variants (ie, one for each subgroup), describing the types of discrepancies and the specific information provided by the search engines for them compared to existing variant specific data.

**Reviewer #3**

The article is well written and best serves biocurators faced with the daunting task to collect specific variant information for the most informed pathogenicity assessment. The authors address the retrievability and segmentation of important information used for the expert review of the clinical significance of potentially disease-causing variations, the numbers of which have drastically increased. The nature of discordance amongst variant classifications is of importance to a broader audience as are the tools available. This is of particular utility for Lynch syndrome variant classification as thorough understanding of the spectrum and complexity of variant information already exists through ongoing variant pathogenicity classification efforts while the dangers of misclassification or ‘diagnostic limbos’ remain a concern.  

The authors compare the article yield, relevance and uniqueness obtained by two search approaches (Google Scholar and Mastermind) of 80 variants, showing that while both methods retrieve relevant unique articles, Mastermind did so more frequently. The authors show that literature searching tools can add incremental value to manual curation, to help decrease classification discordance or absence across a set of MMR variants. While facilitating this process is extremely valuable in helping reduce variant classification discordances, the link to delivering the promise of precision medicine is perhaps not within the deliverables of literature searching tools without further discussion.  

More specific comments: 

3.1 Although it is clear that both methods resulted in unique relevant articles and hence complement each other well, it is unclear why the authors conclude Mastermind to play a complementary role to the standard Google Scholar (control), not the other way around (although discussed). While both search methods missed some information, does the higher number of search results by Google Scholar outweigh the higher number of relevant articles with a higher number and percentage of unique articles yielded by Mastermind or has this been misunderstood? 

3.2 For clarity, it would be interesting to include the authors affiliation or lack of it to the chosen commercial literature searching tool. 

3.3 Duplicate search results were included only once, but when assessing articles for relevance, were duplicate findings also ruled out (ie. Original articles included only?)

3.4 If feasible, it would be interesting to know whether articles not found by either method for 10 variants exist in English.

3m1 Page 8. Second last line: “towas”

3m2 There are no ethical concerns or lack of analysis methods etc.

| 2^nd^ Editorial Decision 13-Jan-2021 |
| --- |

We have now decided to accept the revised manuscript in principle, subject to the attached formatting requirements.
